# Poor clinical response in rheumatoid arthritis is the main risk factor for diabetes development in the short-term: A 1-year, single-centre, longitudinal study

**DOI:** 10.1371/journal.pone.0181203

**Published:** 2017-07-12

**Authors:** Piero Ruscitti, Francesco Ursini, Paola Cipriani, Vasiliki Liakouli, Francesco Carubbi, Onorina Berardicurti, Giovambattista De Sarro, Roberto Giacomelli

**Affiliations:** 1 Division of Rheumatology, Department of Biotechnological and Applied Clinical Sciences, University of L'Aquila, L'Aquila, Italy; 2 Department of Health Sciences, University of Catanzaro “Magna Graecia”, Catanzaro, Italy; Keio University, JAPAN

## Abstract

**Objectives:**

Despite of the European League Against Rheumatism (EULAR) provided different sets of recommendations for the management of cardiovascular risk in inflammatory arthritis patients, it must be pointed out that cardiometabolic comorbidity, such as type 2 diabetes (T2D), remains still underdiagnosed and undertreated in patients affected by rheumatoid arthritis (RA).

**Methods:**

In this work, we designed a single centre, prospective study in order to better investigate the occurrence of T2D during the course of 1 year of follow-up. Furthermore, we evaluated the role of both traditional cardiovascular and RA-specific related risk factors to predict the occurrence of new T2D.

**Results:**

In this study, we evaluated 439 consecutive RA patients and we observed that 7.1% of our patients (31/439) developed T2D, after 12 month of prospective follow-up. The regression analysis showed that the presence of high blood pressure, the impaired fasting glucose (IFG) at the first observation and the poor EULAR-DAS28 response, after 12 months of follow–up, were significantly associated with an increased likelihood of being classified as T2D. Similarly, we observed that 7.7% of our patients (34/439) showed IFG after 12 months of prospective follow-up. The regression analysis showed that the presence of high blood pressure and the poor EULAR-DAS28 response after 12 months of follow-up, were significantly associated with an increased likelihood of showing IFG.

**Conclusions:**

Our study supports the hypothesis of a significant short-term risk of T2D in RA patients and of a close associations between uncontrolled disease activity and glucose metabolism derangement. Further multicentre, randomised-controlled studies are surely needed in order to elucidate these findings and to better ascertain the possible contribution of different therapeutic regimens to reduce this risk.

## Introduction

Rheumatoid arthritis (RA) is a systemic, inflammatory, autoimmune disorder, mainly affecting the joints and associated with a reduction of quality of life [1]. During RA, the therapeutic strategies are aimed at preventing joint destruction by using synthetic disease modifying antirheumatic drugs (sDMARDs) as well as biologic agents [2–5]. In addition, a growing body of evidence is focused on the development of associated comorbidities and their management in rheumatic patients [6–8]. In this context, it has been shown that a large percentage of RA patients can be affected by T2D, as reported by meta-analytic data [9,10]. This clinical phenotype may result from a sinergy between an elevated prevalence of traditional risk factors and pro-inflammatory milieu [11,12]. Furthermore, some well-known pathogenic pro-inflammatory mediators in RA, such as interleukin-1β (IL-1β) and tumor necrosis factor (TNF), may play a pivotal role in the development of T2D, contributing to beta-cells disfunction and distruction and insulin resistance as observed in bone damage [13,14]. To date, early reports suggest that biologic agents commonly used to treat RA patients may be effective in controlling comorbid T2D in both preclinical and clinical settings [13–17].

Despite of the European League Against Rheumatism (EULAR) provided different sets of recommendations for the management of cardiovascular risk in inflammatory arthritis patients [18,19], cardiometabolic comorbidity remains still underdiagnosed and undertreated in RA patients [20]. Although these comorbidities are frequently occurring in RA patients, the evidence deriving from randomized clinical trials does not fully elucidate this topic. In fact, due to strict enrollement criteria usually characterizing the trials, the involved patients may not mirror the real clinical scenario seen during the daily practice, thus significantly decreasing the generalisability of the results [21]. Furthermore, available data in the evaluation of cardiometabolic comorbitities are mainly obtained from low quality studies, generally retrospective or cross sectional, or alternatively from medical records and registers. Different biases, such as selection, reporting and recall biases my be commonly recognized in such studies, impairing the derived results. In addition, a comphensive evaluation of metabolic status in the rheumatologic setting could be complex, expensive and time-consuming and thus the identification of biomarkers accurately reflecting the cardiometabolic risk profile are still awaited [22–24].

In this work, we designed a single centre, prospective study in order to better investigate the occurrence of T2D during the course of 1 year of follow-up. Furthermore, we evaluated the role of both traditional cardiovascular and RA-specific related risk factors to predict the occurrence of new-onset T2D.

## Materials and methods

### Study design and patients

**Fig 1:**
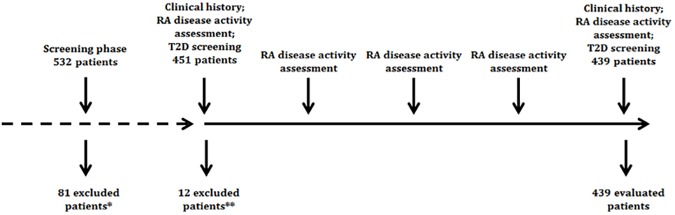
Study design. * In the pre-recruitment screening phase, patients were excluded if presented: 1) past diagnosis of T2D previously performed by a physician *or* 2) current or past treatment with antidiabetic medications (including oral antidiabetic drugs and insulin); 3) fasting plasma glucose (FPG) ≥ 126 mg/dL in at least two separate occasions. ** In the recruitment screening phase patients were excluded if fasting plasma glucose (FPG) ≥ 126 mg/dL.

The present study was designed as a 12-months longitudinal observational cohort study aimed at evaluating the risk of development of T2D in RA patients (Fig 1). For this purpose, all consecutive newly-diagnosed RA patients attended our Department during a 5-years period (January 2011 –December 2015) were screened for eligibility. RA patients were classified according to 2010 American College of Rheumatology (ACR)/EULAR and/or 1987 ACR criteria [25,26]. Exclusion criteria were predefined as follows: 1) past diagnosis of T2D previously performed by a physician *or* 2) current or past treatment with antidiabetic medications (including oral antidiabetic drugs and insulin); 3) fasting plasma glucose (FPG) ≥ 126 mg/dL in at least two separate occasions. In this study, we strictly followed the international and national recommendations for the follow up and treatment of RA patients. In our cohort, according to the EULAR recommendations, the management of patients was governed by a strategic therapeutic approach aimed at remission or at low disease activity. On the way to attaining these targets, patients were closely monitored using DAS28(ESR) and treatment adapted in accordance [27].

The local ethics committee (*Azienda Sanitaria Locale 1 Avezzano/Sulmona/L’Aquila*, *L’Aquila*, *Italy*) approved this study. It has been performed according to the Good Clinical Practice guidelines, and written informed consent was obtained from all patients, according to the Declaration of Helsinki [28].

### Study protocol

The study protocol is summarized in Fig 1.

Briefly, after the screening phase, 81 patients were excluded because presenting one or more of the exclusion criteria. The remaining patients (n = 451) underwent baseline assessment, including clinical evaluation and screening for the presence of T2D according to American Diabetes Association (ADA) 2009 recommendations [29]. All patients with FPG ≥ 126 mg/dL (n = 12) were excluded. The remaining patients (n = 439) entered the 12 months follow-up. Treatment for RA was started according to clinical judgement in respectful of local treatment guidelines and disease activity was assessed at three months intervals (see below) according to tight control RA recommendations [27]. Therapy was liberally modified during the observation period according to clinical judgement. At the end of the observation period, patients were reassessed for the presence of diabetes according to prespecified criteria stated under the “definition of cases subheading” (see below).

### Definition of cases

Screening for T2D was performed at baseline and after 12 months according to ADA 2009 recommendations [28]. Briefly, patients underwent measurement of plasma glucose after overnight fasting. If FPG was < 100 mg/dL, patients were classified as normal glucose tolerance (NGT). If FPG was 100 to 125 mg/dL patients were classified as having impaired fasting glucose (IFG). If FPG ≥ 126 mg/dL patients were classified as having T2D. Abnormal results (≥ 100 mg/dL) were reassessed one week later. If there was inconsistency between the two values, a third evaluation was performed after an additional week and the category assignment was performed according to the consistency between two out of three values. At the month 12 follow-up visit, in addition to the above FPG-based classification, patients were classified as T2D also if FPG < 100 mg/dL but presenting one or more of the following criteria: 1) diagnosis of T2D performed during the observation period by a physician (i.e. general practicioner) or 2) therapy with antidiabetic medications (including oral antidiabetic drugs and insulin) prescribed by a physician.

### Clinical history and disease activity assessment

Relevant data were collected at study entry and reassessed at the end of the follow-up period including demographic characteristics, smoking habit, RA features and complications, comorbidities and medications used. The presence of obesity was assessed by using the body mass index (BMI) to cathegorise patients as underweight (< 18.5 kg/m^2^), normal weight (18.5–25 kg/m^2^), overweight (25–30 kg/m^2^) or obese (> 30 kg/m^2^). Radiographic damage was defined as the presence of at least one marginal erosion on previously performed hand radiography. For those patients who underwent sequential treatment with DMARDs or biologic agents during the follow-up period, we assigned the treatment category according to the molecule to which the patient was exposed for a longer period. Corticosteroid (CCS) treatment was codified in categories; the first category included patients taking ≥5 mg prednisone-equivalent for 3 months or more during the observation period; the second category included those who took < 5 mg prednisone-equivalent for more than 3 months or ≥ 5 mg prednisone-equivalent for less than 3 months; the third category included patients who did not take corticosteroids during the follow-up.

The Disease Activity Score including 28 joints (DAS28-ESR) was used to assess disease activity, evaluating the number of swollen joints (SJC), number of tender joints (TJC) and erythrocyte sedimentation rate (ESR, mm/h). ESR was included in the assessment of DAS28 in order to maintain the independence of CRP, a well-estabilished cardiometabolic risk factor in the general population [30] for data analysis. At the end of the study period, serial DAS28 measurements were used to evaluate treatment efficacy according to European League Against Rheumatism (EULAR) response criteria [31].

### Laboratory evaluation

Blood samples were obtained for laboratory evaluation after overnight fasting. FPG was measured with an automated chemistry analyzer (Cobas 6000/Cobas e411, Roche Diagnostics). Erythrocyte sedimentation rate (ESR) was evaluated by capillary photometry (Test 1, Alifax). High sensitivity C-reactive protein was measured by immunonephelometry (CardioPhase® hsCRP, Siemens HealthCare). Rheumatoid factor (RF) was analyzed by nephelometry (BN II system, Siemens HealthCare). Anti-cyclic citrullinated peptide antibodies (ACPA) were analysed with chemiluminescent immunoassay (Zenit RA CCP, Menarini Diagnostics).

### Statistical analysis

Data are expressed as mean ± standard deviation, median (25th–75th percentile), or number (percentage) as appropriate. Continuous variables that were not normally-distributed were *ln*-transformed before analysis. Single- and multiple- logistic regression models were built in order to evaluate the contribution of selected variables on the likelihood of developing T2D or IFG. Multicollinearity between independent variables was evaluated by using the variance inflation factor (VIF) before entering each value in the regression model. Receiver operating characteristic (ROC) curves were built to evaluate the predictivity of mean DAS28 on the likelihood of having T2D or IFG. The best cut-off for ROC curves was calculated with the Youden’s index [32] as previously described. A p-value < 0.05 was considered statistically significant. All tests were two-tailed. The Statistics Package for Social Sciences (SPSS for Windows, version 17.0, SPSS Inc., Chicago, IL, USA) was used for all analyses.

## Results

### Baseline clinical characteristics

In this study, we evaluated 439 consecutive RA patients, fulfilling 2010 ACR/EULAR and/or 1987 ACR Criteria. The baseline clinical characteristics are reported in Table 1. The majority of our patients showed a seropositive disease (81.7%) with a mean duration of 5.1±3.8 years. Thirteen percent of our patients were affected by different extra-articular complications. Methotrexate (MTX) and TNF inhibitors (TNFis) were the most common medications prescribed during the study period. The mean value of FPG at the beginning of the study was 89.6 ± 14.1 mg/dL with 18% of patients fulfilling the criteria for IFG.

**Table 1:** Demographic and clinical characteristics of the study cohort.

Descriptive statistics	
Female (male)	378 (61)
Age, years (mean ± SD)	58.38±13.50
Smoking habit, number, percentage	151, 29.8%
Hypertension, number, percentage	188, 37,1%
BMI category, number, percentage	
<18.49	20, 3.9%
<18.5<24.99	258, 50.9%
>25<29.99	122, 24.1%
>30	39, 7.7%
Fasting glucose t0, mg/dl (mean ± SD)	89.6±14.1
Fasting glucose t12, mg/dL (mean ± SD)	92.2±15.8
IFG t0, number, percentage	92, 18.1%
RA duration, years (mean ± SD)	5.1±3.8
RF+ve and/or ACPA+ve, number, percentage	359, 81.7%
Extra-articular features, number, percentage	69, 13.6%
Past joint surgery, number, percentage	33, 6,5%
DAS28 (ESR) t0, (mean ± SD)	4.86±0.99
DAS28 (ESR) t12, (mean ± SD)	2.64±1.35
DAS28 Remission t0 <2.6, percentage	0.6%
DAS28 Low disease activity t0 ≥2.6 ≤3.2, percentage	1.6%
DAS28 Moderate disease activity >3.2 ≤5.1, percentage	49.7%
DAS28 High disease activity >5.1, percentage	34.7%
EULAR poor response t12, percentage	8.5%
EULAR moderate response t12, percentage	23.7%
EULAR good response t12, percentage	54.5%
HAQ t0, (mean ± SD)	0.9±0.6
HAQ t0, (mean ± SD)	0.5±0.6
CRP t0, mg/L (mean ± SD)	8.9±14.4
CRP t12, mg/L (mean ± SD)	1.9±3.6
Radiographic damage t0, number, percentage	100, 19.7%
MTX-treated*, number, percentage	367, 72.4%
CCS ≥ 5 mg-treated, number, percentage	355, 80.9%
CCS < 5 mg-treated, number, percentage	30, 6.8%
TNFi-treated*, number, percentage	179, 35.3%
Other Biologics-treated*, number, percentage	55, 10.8%

BMI, body mass index; IFG, impaired fasting glucose; RA, rheumatoid arthritis; RF, rheumatoid factor; ACPA, anti-cyclic citrullinated peptide antibodies; DAS28 (ESR), disease activity score including 28 joints and erythrocyte sedimentation rate; EULAR, European League Against Rheumatism, HAQ, health assessment questionnaire; CRP, C-reactive protein; MTX, methotrexate; CCS, corticosteroids; TNFi, TNF inhibitors. * Assignment to the treatment category was performed according to the medication used for the longest period during the whole follow-up duration.

### Follow-up

The enrolled patients were evaluated every 3 months for assessment of RA disease activity. During the follow-up, a progressive reduction of both DAS28(ESR) and HAQ have been observed. At month 12, DAS28(ESR) was 2.64 ± 1.35 and HAQ was 0.53 ± 0.60, respectively. We observed that 49.65% of patients (218/439) reached DAS28 remission (<2.6) and 13.66% reached low disease activity (60/439). Moreover, we assessed the mean value, calculated from the different visits thoughtout the follow-up, of DAS28(ESR), HAQ and *ln*CRP in order to estimate the burden of disease activity in addition to the time-point evaluation at the end of the follow-up. At month 12, mean DAS28(ESR) was 3.22 ± 1.16, mean HAQ was 0.62 ± 0.57 and *ln*CRP was 1.04 ± 0.86 mg/L, respectively. The mean value of DAS28(ESR), during the follow up, was significantly higher in patients taking ≥ 5 mg prednisone-equivalent for 3 months or more during the observation period when compared with the other patients (2.75 ± 1.32 vs 2.17 ± 1.39, p < 0.0001). We observed that 54.4% of the enrolled patients reached a good clinical response according to EULAR criteria at the end of follow-up, whereas 8.5% out of patients did not. Radiographic progression was observed in 15.4% of patients, at month 12.

### Development of T2D and predictors

At the end of the follow-up, at month 12, screening for T2D was performed as done at the beginning of the study. We observed that 7.1% of our patients (31/439) developed T2D according to the established criteria. A logistic regression model was perfomed to evaluate the possible predictive role of selected variables [High blood pressure, poor EULAR-DAS28(ESR) response at month 12, HAQ mean, *ln*CRP, radiographic progression] on the likelihood that patients developed T2D. No significant multicollinearity between the selected variables was observed.

The logistic regression model was statistically significant (χ2 = 79.32, p < 0.0001). The analysis showed that the presence of high blood pressure, IFG and poor EULAR-DAS28(ESR) response at month 12 were associated with an increased likelihood of being classified as T2D. The presence of high blood pressure was associated with 6.83 fold higher risk of having T2D (OR = 6.83, 95% CI 2.18–21.34, p = 0.001) and the presence of IFG at the study entry was associated with a risk of 30.55 (OR = 30.55, 95% CI 6.53–142.76, p < 0.0001). The poor EULAR-DAS(28) response at month 12 was associated with 33.598 fold higher risk of having T2D (OR = 33.59, 95% CI: 6.95–162.21, p < 0.0001). In our model, the mean values of HAQ during the follow up, the mean *ln*CRP, and the development of radiographic progression did not seem to be associated with the risk to develop T2D. The regression analyses are summarized in Table 2.

**Table 2:** Logistic regression analysis for prediction of new-onset type 2 diabetes.

	Univariate analyses				Multivariate analysis			
Variable	OR	Lower	Upper	P	OR	Lower	Upper	P
Gender	1.540	0.605	3.924	0.365				
Age	1.024	0.995	1.054	0.100				
**Hypertension**	**7.896**	**2.971**	**20.985**	**<0.0001**	**6.832**	**2.187**	**21.349**	**0.001**
BMI>30	1.107	0.321	3.821	0.872				
**IFG_t0**	**3.484**	**1.647**	**7.370**	**<0.0001**	**30.555**	**6.539**	**142.765**	**<0.0001**
RA duration	1.030	0.985	1.077	0.199				
RF+ve	0.504	0.242	1.051	0.068				
ACPA+ve	0.515	0.237	1.121	0.095				
Extra-articular features	1.979	0.846	4626.000	0.115				
Surgery	0.392	0.052	2.967	0.364				
Smoke	1.053	0.491	2.259	0.895				
DAS28_t0	0.809	0.555	1.179	0.270				
**DAS28_t12**	**2.237**	**1.583**	**3.160**	**<0.0001**				
**Poor EULAR response**	**12.723**	**5.704**	**28.378**	**<0.0001**	**33.598**	**6.959**	**162.213**	**<0.0001**
**DAS28_mean**	**1.950**	**1.340**	**2.838**	**<0.0001**				
HAQ_t0	0.921	0.504	1.685	0.790				
**HAQ_t12**	**3.343**	**1.875**	**5.961**	**<0.0001**				
**HAQ_mean**	**2.215**	**1.233**	**3.980**	**0.008**	1.098	0.486	2.477	0.822
CRP_t0	1.006	0.985	1.027	0.577				
**CRP_t12**	**1.165**	**1.091**	**1.244**	**<0.0001**				
**lnCRP_mean**	**1.921**	**1.234**	**2.991**	**0.004**	1.052	0.986	1.123	0.123
Radiographic damage_t0	1.677	0.762	3.690	0.199				
**Radiographic damage_t12**	**4.673**	**2.193**	**9.957**	**<0.0001**				
**Radiographic progression**	**2.799**	**1.282**	**6.112**	**0.010**	1.820	0.695	4.768	0.223
MTX	1.330	0.451	3.926	0.605				
CCS ≥ 5 mg	3.771	0.882	16.122	0.073				
CCS < 5 mg	0.298	0.040	2.245	0.240				
TNFi	1.603	0.771	3.333	0.206				
Other Biologics	0.505	0.117	2184.000	0.361				

BMI, body mass index; IFG, impaired fasting glucose; RA, rheumatoid arthritis; ACPA, anti-cyclic citrullinated peptide antibodies; DAS28, disease activity score including 28 joints; EULAR, European league against rheumatism; HAQ, health assessment questionnaire; CRP, C-reactive protein; MTX, methotrexate; CCS, corticosteroids; TNFi, TNF inhibitors.

Given the well-known prodiabetogenic effect of CCS, we performed a separate multivariate analysis including CCS treatment as predictor, in addition to the above mentioned variables, despite CCS was not significantly associated with T2D development in univariate analysis. In this additional model, poor EULAR response (OR = 35.46, 95% CI 6.99–179.67, p < 0.0001), high blood pressure (OR = 9.03, 95% CI 2.64–30.81, p < 0.0001), lnCRP (OR = 1.85, 95% CI 1.04–3.29, p = 0.03) and baseline IFG (OR = 29.33, 95% CI 6.14–140.08, p < 0.0001) maintained their strong correlation with T2D while no association was found with CCS treatment (OR 3.22; 95% CI 0.58–17.95, p = 0.18).

**Fig 2:**
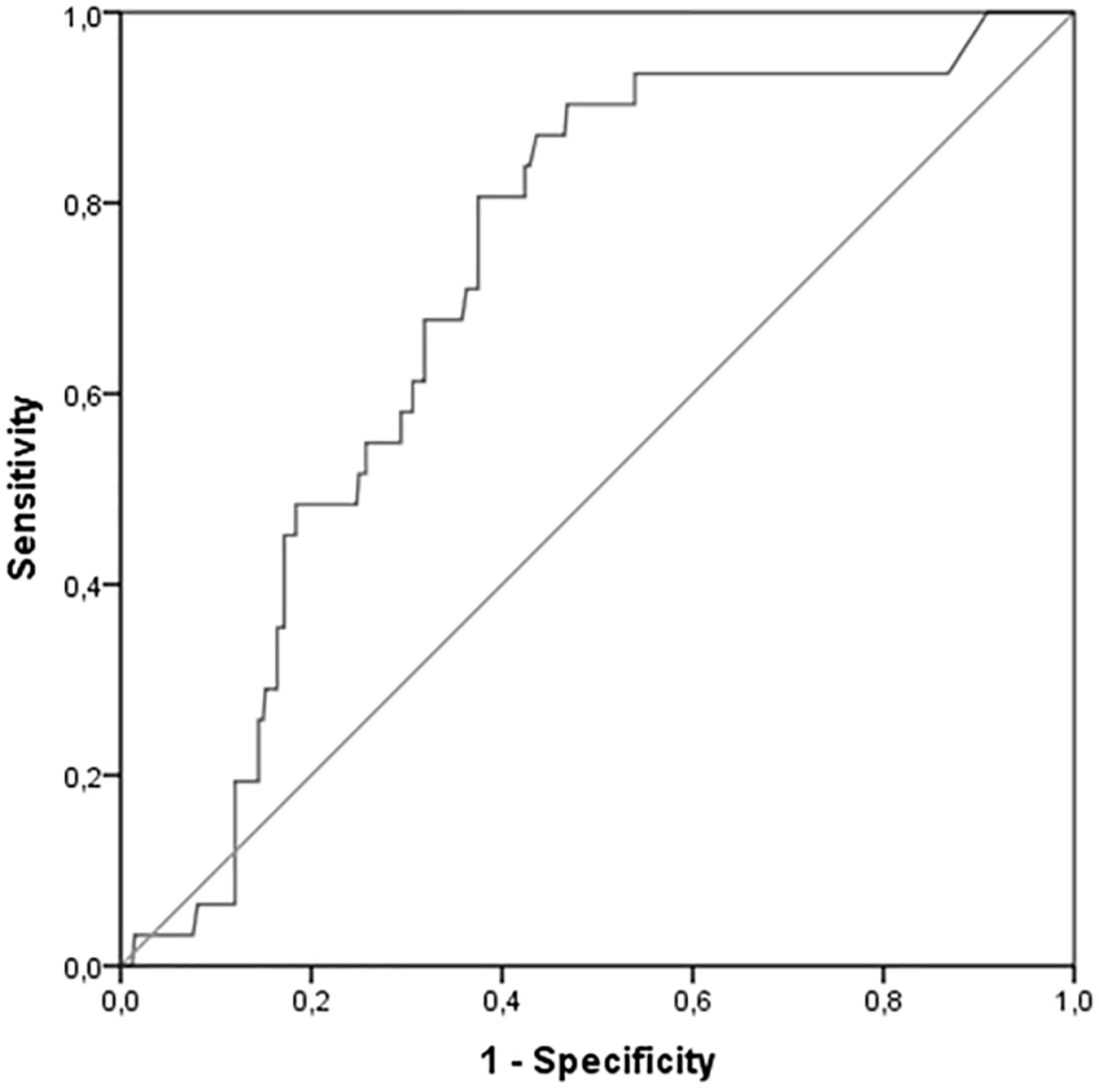
Receiver operator characteristic (ROC) curve of mean value DAS28(ESR) in predicting the development of type 2 diabetes (T2D).

Furthermore, we performed a ROC curve in order to investigate the predictivity of mean value of DAS28(ESR) on the likelihood of being diagnosed with T2D (Fig 2). The area under the ROC curve was 0.79 (95% CI: 0.63–0.79, p < 0.0001) for the mean value of DAS28(ESR). The analysis of ROC curve showed that the the best cut-off for mean DAS28(ESR) was 3.71 and provided a sensitivity of 80% and a specificity of 63%.

### Development of IFG and predictors

At the end of the follow-up, at month 12, the possible development of IFG was also investigated. We observed that 7.7% out of our patients (34/439) showed IFG according to the established criteria. A logistic regression model was perfomed to evaluate the possible predictive role of selected variables [Hypertension, BMI>30, no EULAR-DAS28(ESR) response at month 12, HAQ mean, radiographic progression] on the likelihood that patients showed IFG. No significant multicollinearity between the selected variables was observed.

The logistic regression model was statistically significant (χ2 = 128.73, p < 0.0001). The analysis showed that the presence of high blood pressure and the poor EULAR-DAS28(ESR) response at month 12 were associated with an increased likelihood of developing IFG. The presence of hypertension was associated with 5.71 fold higher risk of having IFG (OR = 5.71, 95% CI 1.40–23.27, p = 0.015). The lack of EULAR-DAS(28) response at month 12 was associated with 75.22 fold higher risk of having T2D (OR = 75.22, 95% CI: 23.33–242.72, p < 0.0001). In this model, the presence of BMI>30, the mean values of HAQ during the follow up and the development of radiographic progression did not seem to be associated with the presence of IFG. The regression analyses are shown in Table 3.

**Table 3:** Logistic regression analysis for prediction of new-onset impaired fasting glucose.

	Univariate analyses				Multivariate analysis			
Variable	OR	Lower	Upper	P	OR	Lower	Upper	p
Gender	0.367	0.086	1,570	0.176				
Age	1.017	0.990	1,045	0.225				
**Hypertension**	**16.323**	**4.907**	**54.292**	**<0.0001**	**5.711**	**1.401**	**23.277**	**0.015**
BMI>30	**4.500**	**1.928**	**10.505**	**0.001**	1.624	0.431	6.126	0.474
**Glycemia_t0**	1.011	0.986	1.037	0.377				
RA duration	1.037	0.994	1.082	0.092				
RF+ve	0.606	0.298	1.230	0.165				
ACPA+ve	0.680	0.331	1.395	0.293				
Extra-articular features	0.697	0.238	2.047	0.512				
Surgery	0.754	0.173	3.295	0.708				
Smoke	0.564	0.249	1.278	0.170				
**DAS28_t0**	**0.458**	**0.302**	**0.695**	**<0.0001**				
**DAS28_t12**	**3.644**	**2.390**	**5.554**	**<0.0001**				
**Poor EULAR response**	**121.333**	**43.688**	**336.973**	**<0.0001**	**75.225**	**23.332**	**242.727**	**<0.0001**
**DAS28_mean**	**2.006**	**1.394**	**2.885**	**<0.0001**				
HAQ_t0	1.108	0.639	1.922	0.714				
**HAQ_t12**	**2.639**	**1.528**	**4.558**	**<0.0001**				
**HAQ_mean**	**1.969**	**1.120**	**3.461**	**0.019**	0.738	0.285	1.911	0.532
CRP_t0	0.990	0.956	1.024	0.544				
**CRP_t12**	**1.111**	**1.046**	**1.181**	**0.001**				
**lnCRP_mean**	**1.157**	**0.741**	**1.807**	**0.520**				
Radiographic damage_t0	1.693	0.795	3.605	0.172				
**Radiographic damage_t12**	**2.191**	**1.075**	**4.467**	**0.031**				
**Radiographic progression**	**2.413**	**1.123**	**5.184**	**0.024**	0.941	0.289	3.071	0.920
MTX	0.482	0.214	1.086	0.078				
CCS	0.506	0.231	1.108	0.088				
TNFi	0.746	0.360	1.550	0.433				
Other Biologics	0.000	0.000		0.997				

BMI, body mass index; IFG, impaired fasting glucose; RA, rheumatoid arthritis; ACPA, anti-cyclic citrullinated peptide antibodies; DAS28, disease activity score including 28 joints; EULAR, European league against rheumatism; HAQ, health assessment questionnaire; CRP, C-reactive protein; MTX, methotrexate; CCS, corticosteroids; TNFi, TNF inhibitors.

**Fig 3:**
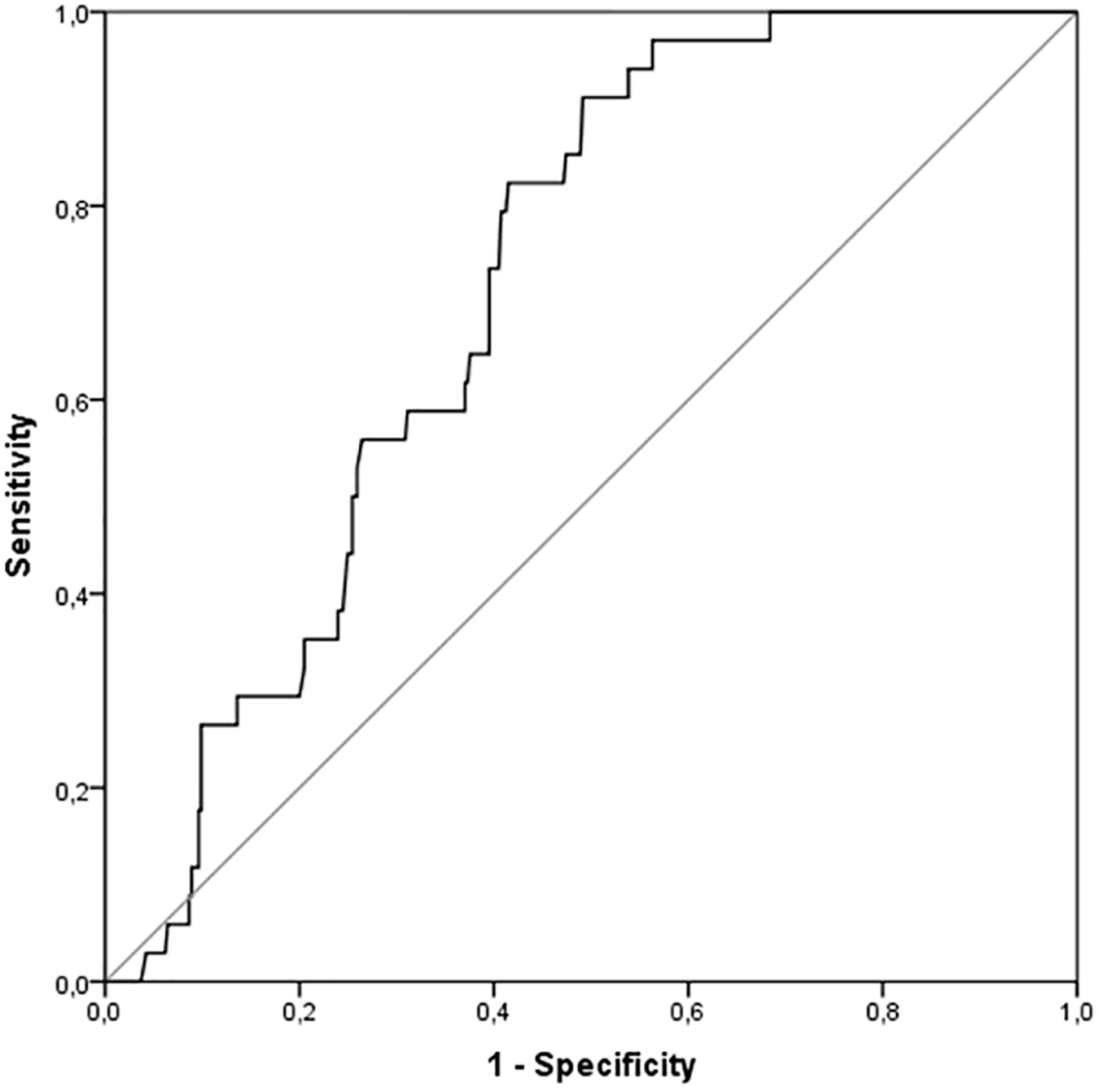
Receiver operator characteristic (ROC) curve of mean value DAS28(ESR) in predicting the development of type 2 diabetes (T2D).

In addition, we performed a ROC curve in order to investigate the predictivity of mean value of DAS28(ESR) on the likelihood of developing IFG (Fig 3). The area under the ROC curve was 0.71 (95% CI: 0.64–0.77, p < 0.0001) for the mean value of DAS28(ESR). The analysis of ROC curve showed that the the best cut-off for mean DAS28(ESR) was 3.30 and provided a sensitivity of 91% and a specificity of 51%.

## Discussion

Several studies showed the close relationship between RA and risk of cardiovascular disease (CVD) to a similar extent of that observed in T2D [33,34]. RA and T2D are independent risk factors for CVD and, therefore, patients affected by both these diseases may be considered at very higher risk of CVD and related mortality [10]. On this background, identifying risk factors for T2D, which shows and increased incidence and prevalence during RA [10], could represent an attractive strategy contributing to overall CVD risk reduction and management of RA patients. On these bases, we planned the present study in order to evaluate the occurrence of new-onset T2D in RA patients in the short-term and to identify the possible predictors of T2D development, thus suggesting a possible synergistic interaction in determining a worse cardiovascular phenotype of RA patients with comorbid T2D [33–36].

According to our data, a high proportion (7.1%) of patients in this single-centre cohort developed T2D despite a short follow-up period; given the limitation due to the lack of an appropriate control group this figure appears significantly higher when compared to the yearly incidence of T2D reported in recent large scale administrative health database from Italy of 4/1000 [37] and, to date, it seems comparable to the incidence associated with IFG, as shown in a recent meta-analysis [38].

In addition, we reported the baseline prevalence of IFG in RA patients and the risk factors for its development after 12 months. IFG, together with impaired glucose tolerance (IGT), represents a transitional abnormality during the natural history of T2D [39]. In contrast to IGT, whose diagnosis requires oral glucose tolerance test (OGTT), IFG is a powerful cardiometabolic risk factor that may be easily identified with a simple measurement of fasting glucose [40]. Few data about IFG in RA are available and, to our knowledge, only two studies evaluated the prevalence IFG in RA patients; the study by Ursini et al. reported a prevalence of about 9% in an Italian RA cohort [41]; a Chinese study reported an higher prevalence of 16% [42]. This figure, however, seems quite in line with the prevalence in the Italian general population that is estimated around 11% [43].

Furthermore, we investigated possible predictors of the short-term development of T2D in our population. Baseline IFG was a strong predictor of T2D as well as the presence of high blood pressure. In fact, high blood pressure is a well-established risk factor for T2D in the general population independently of the medications used to lower blood pressure [44]. In addition, several measures of disease activity, and in particular a poor EULAR response at month 12, were associated with the development of T2D in univariate analysis, and this correlation was maintained in a multivariate model including baseline IFG and high blood pressure. Collectively, our data support the hypothesis that failure to reach a EULAR-defined moderate or good response is a major risk factor for the development of T2D in the short term, with a strength of association supporting the growing theory that CVD risk in RA results from a detrimental sinergy between inflammation and traditional cardiovascular risk factors [45–47]. Similarly to what observed for T2D, the poor clinical response was associated with the risk of the development of IFG at the end of the observation period, together with other predictors including BMI and hypertension. Moreover, we performed ROC curves investigating the role of mean DAS28 in predicting T2D and IFG. According to our analysis, a DAS28(ESR) of 3.30 and 3.71 were selected as best cut-off respectively for predicting T2D and IFG development, thus supporting the concept that also a disease activity in the “moderate” range may significantly affect the risk of worsening in glucose tolerance. Our data suggest the growing concept that RA patients must be treated as intensively as possible aiming at remission or, at least, low-disease activity in order to minimise the detrimental effect or inflammation on cardiometabolic profile.

Surprisingly, in contrast with previously published data supporting a protective role of TNFi [48], in our population the risk of T2D was independent of treatment with TNFi or steroids. This discrepancy, in our opinion, could be the result of a low power of our study to catch a significant effect of TNFi or, from the other side, of a higher probability of those patients treated with TNFi to reach a good EULAR response. In addition, due to the specific real-life design of our study, in which the treatments of RA patients were not randomised, any possible association between the treatments and the outcomes may be impaired by different biases [49,50]. In particular, in our cohort we failed to confirm the already well-recognized prodiabetogenic effect of CCS. However, the design of our study implied that CCS dosage was frequently modified according to the unconditioned clinical judgement of the physician; for this reason it was impossible to calculate with sufficient precision the cumulative exposure to CCS of individual patients and cathegorization of CCS treatment was, in our opinion, the best alternative surrogate measure of CCS exposure. In this context, it must be pointed out that this represents one of the main limitations of our study and generalisation of the effect of treatment regimens on the development of T2D to the whole RA population need to be further investigated.

Taken together, our data may suggest the possible pathogenic link, already observed in other studies, that treatment failure or prolonged periods of uncontrolled disease activity, underlying an active inflammatory process, represent a strong contributor to cardiometabolic risk in RA patients [12,51,52]. Interestingly, treatment response per se, independently of molecule used to achieve it, may represent the strongest determinant of cardiometabolic risk in the short-term [53,54]. In this view, a better management of persistent poorly controlled RA patients, with conventional therapies and biologic drugs, could confer additional benefits on the joint damage as well as on cardiometabolic comorbidity, thus improving long-term outcome [55–57].

Despite providing a deeper insight into cardiometabolic risk associated with RA, our study has different limitations. The lack of a control group avoid to quantify the relative risk, conferred by RA, of new-onset T2D and IFG when compared with randomly selected individuals from the general population. In addition, the short follow-up period and the original study design, aimed at investigating a specific cardiometabolic outcome, did not allow to ascertain the possible contribution of glucose tolerance worsening on hard cardiovascular outcomes such as the number of major cardiovascular events. Moreover, we did not perform OGTT, the only technique that allow to recognise the presence of IGT, and thus we probably misclassified a small subset of IGT patients as normal, relying only on the absence of T2D and IFG. However, performing OGTT is complex and time-consuming in the rheumatologic setting and may expose the patients to adverse events like nausea, dizziness and severe hypoglycaemia [58]. Finally, the single centre design, although limiting classification bias, may reduce the external validity of our findings.

In conclusion, our study supports the hypothesis of a significant short-term risk of T2D in RA patients and of a close associations between uncontrolled disease activity and glucose metabolism derangement. Further multicentre, randomised-controlled studies are surely needed in order to elucidate this finding and to better ascertain the possible contribution of different therapeutic regimens to reduce this risk.
